# Elevation and phylogeny shape herbaceous seed dormancy in a biodiversity hotspot of southwest China

**DOI:** 10.1002/ece3.9986

**Published:** 2023-04-18

**Authors:** Kai Chen, Zi‐Hong Chen, Yuan‐Yuan Huang, Zhong‐Hua Jiang

**Affiliations:** ^1^ Construction Projects about the Key Laboratory of Entomogenous Fungi Resources Conservation and Green Development in Gaoligong Mountains Baoshan University Baoshan Yunnan 678000 China; ^2^ Research Institute of Gaoligong Mountains Baoshan University Baoshan Yunnan 678000 China; ^3^ School of Resources and the Environment Baoshan University Baoshan Yunnan 678000 China

**Keywords:** climatic factors, elevation, *Impatiens* species, phylogeny, seed dormancy status

## Abstract

Seed dormancy contributes greatly to successful establishment and community stability and shows large variation over a continuous status scale in mountain ecosystems. Although empirical studies have shown that seed dormancy status (SDS) is shaped by elevation and phylogenetic history in mountain ecosystems, few studies have quantified their combined effects on SDS. Here, we collected mature seeds from 51 populations of 11 *Impatiens* species (Balsaminaceae) along an elevational gradient in the Gaoligong Mountains of southwest China and estimated SDS using mean dormancy percentage of fresh seeds germinated at three constant temperatures (15, 20, and 25°C). We downloaded 19 bioclimatic variables from WorldClim v.2.1 for each *Impatiens* population and used internal transcribed spacer (ITS), *atpB*‐*rbcL*, and *trnL‐F* molecular sequences from the GenBank nucleotide database to construct a phylogenetic tree of the 11 species of *Impatiens*. Logistic regression model analysis was performed to quantify the effects of phylogeny and environment on SDS. Results identified a significant phylogenetic SDS signal in the *Impatiens* species. Furthermore, elevation and phylogeny accounted for 63.629% of the total variation in SDS among the *Impatiens* populations. The best logistic model indicated that temperature was the main factor influencing variation in SDS among the *Impatiens* species, and model residuals were significantly correlated with phylogeny, but not with elevation. Our results indicated that seed dormancy is phylogenetically conserved, and climate drives elevational patterns of SDS variation in mountain ecosystems. This study provides new insights into the response of seed plant diversity to climate change.

## Graphical abstract

This manuscript reveals that seed dormancy is an ecological adaptation strategy and is phylogenetically conserved that provides new insights into the response of seed plant diversity to climate change.

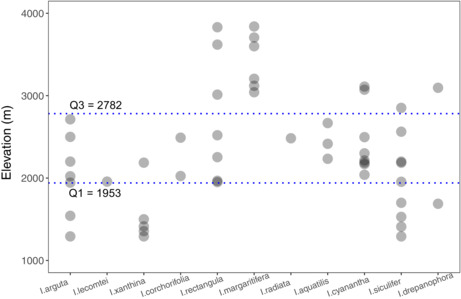

## 1 INTRODUCTION

Seed dormancy, which is defined as the failure of a viable seed to germinate in a suitable environment, can regulate germination timing (Fenner, 2002; Willis et al., 2014). However, it is not an all‐or‐nothing trait, but rather varies widely across a continuous range of seed dormancy status (SDS) (Batlla & Benech‐Arnold, 2007). Not only is it influenced by many genes with small effects but it also changes with the environment of seed maturation (Andersson & Milberg, 1998; Baskin & Baskin, 2004; Finch‐Savage & Leubner‐Metzger, 2006). Seed dormancy, as an adaptive trait of seed plants, plays a critical role in promoting successful establishment and community stability by ensuring suitable germination time through SDS variation, thus avoiding unfavorable conditions for seedling growth, especially in mountain ecosystems (Aragón‐Gastélum et al., 2018; Finch‐Savage & Leubner‐Metzger, 2006; Jurado & Flores, 2005).

Elevation is a typical environmental feature of mountain ecosystems (Sundqvist et al., 2013), and elevation provenance of seeds is an important factor associated with dormancy. Plants distributed over a wide range of elevations exhibit highly variable SDS due to different natural selection pressures (Allen & Meyer, 1998; Jurado & Flores, 2005; Veselá et al., 2020). For example, populations of *Saxifraga longifolia* (Saxifragaceae) from high elevations show the highest proportion of dormant seeds (Cotado et al., 2020), while *Cardiospermum halicacabum* (Sapindaceae) seeds collected from high‐elevation habitats show more complex SDS than seeds from low‐elevation habitats, requiring more periods of cold stratification to break dormancy (Orrù et al., 2012; Thusithana et al., 2021). Low‐elevation populations of wild grapevine (*Vitis vinifera* subsp. *sylvestris*) are threatened by climate warming due to compromised seed dormancy release (Orrù et al., 2012). Furthermore, a positive relationship exists between the number of dormant seeds and elevation in *Physalis* (Solanaceae) species (Farooq et al., 2021). While previous studies have reported on variation in SDS with elevation at the intra‐ or interspecific level, climatic factors, such as temperature and precipitation, are also likely to play important roles in modulating SDS variation with elevation. However, the extent to which microclimatic conditions at different elevations accurately predict SDS remains unclear, necessitating further combined research efforts.

Elevational gradients related to SDS are closely linked to plant adaptation to local climatic conditions (Andersson & Milberg, 1998). Increases in elevation are accompanied by significant changes in climate, including notable declines in temperature. Furthermore, climatic factors experienced during seed maturation, particularly heat and moisture, can exert pronounced effects on seed dormancy (Cotado et al., 2020; Penfield, 2017; Wagmann et al., 2012). Plants under low‐temperature stress produce many more small seeds with high dormancy than plants in normal habitats (Rosbakh et al., 2022). For example, the proportion of dormant seeds in *Beta vulgaris* (Amaranthaceae) populations varies from 0 to 1 under different ambient temperatures (Wagmann et al., 2012). Precipitation is another crucial limiting factor, with seeds developing in dry habitats more prone to dormancy due to adaptive predictive germination (Thusithana et al., 2021). Although changes in climate due to increasing elevation may drive elevational patterns of SDS variation, direct evidence is still lacking.

As closely related species typically experience similar natural selection pressures and seed traits can be constrained by phylogenetic history, species with a common ancestor often exhibit similar SDS (Chen et al., 2022; Seglias et al., 2018). For example, morphological dormancy is considered the ancestral state of seed dormancy and is observed in primitive taxa such as Taxaceae, Ginkgoaceae, and Podocarpaceae (Finch‐Savage & Leubner‐Metzger, 2006). Physical seed dormancy only occurs in angiosperm seeds (e.g., Convolvulaceae), whereas physiological seed dormancy occurs in basal taxa (e.g., Gymnosperms) to the higher core eudicot clade of rosids (Finch‐Savage & Leubner‐Metzger, 2006; Willis et al., 2014). In addition, both physiological and physical dormancy diverged from morphological dormancy, a driving force of lineage diversification of seed plants (Donohue et al., 2005; Willis et al., 2014). Although seed dormancy is phylogenetically constrained, the phylogenetic effects and relationship between phylogenetic distance and seed plant SDS remain unquantified.

The Balsaminaceae genus *Impatiens* L. is one of the largest within angiosperms and is widely distributed at different elevations in mountain ecosystems (Yu et al., 2016). Within temperate mountain ecosystems, *Impatiens* species seeds typically exhibit one of three types of physiological dormancy, including nondeep, intermediate, and deep dormancy (Baskin & Baskin, 2014). For example, *Impatiens biflora* seeds are characterized by nondeep physiological dormancy, while *Impatiens parviflora* seeds are characterized by deep physiological dormancy (Baskin & Baskin, 2014). As such, *Impatiens* species represent an excellent group for investigating SDS variation along elevational gradients. In addition, genus‐specific study allows us to control for important ecological traits associated with seed dormancy (e.g., growth form, dispersal mode, and fruit type), while identifying genuine sources of SDS variation in elevational patterns (Ge et al., 2020).

Environmental selection is the basic driving force of seed trait evolution (Moles et al., 2005), with seed plants colonizing a wide range of elevational habitats and developing a wide range of dormancy strategies during diversification in mountain ecosystems (Moles et al., 2005). Consequently, ecological differentiation along elevational gradients is expected to promote SDS divergence. In the current study, we aimed to quantify the effects of phylogeny and elevation on SDS variation in *Impatiens* species in mountain ecosystems. We predicted that phylogenetic distance would be significantly correlated with SDS, and phylogeny and elevation would jointly explain SDS variation. We investigated the relationship between SDS and phylogenetic and climatic factors in 51 populations of 11 species of *Impatiens* widely distributed in the mountains of southwest China. The following three questions were addressed: (1) Are there significant phylogenetic signals of SDS in *Impatiens* species? (2) How much of the SDS variation in *Impatiens* can be explained by both phylogeny and elevation? (3) After controlling for climatic factors, are the relationships between SDS and phylogeny and elevation still significant?

## 2 MATERIALS AND METHODS

### 2.1 Study area

The Gaoligong Mountains in southwestern China (97°30′–99°30′ E, 24°40′–28°30′ N) are characterized by a huge elevational range and extremely high levels of biodiversity (Chen et al., 2020). The climate is primarily controlled by the Indian Ocean monsoon, with heavy summer precipitation and low winter precipitation. The region also exhibits substantial spatial changes in climatic conditions, with a mean annual temperature of 5.4–14.9°C and mean annual precipitation of 648–1591 mm across elevations (Liang et al., 2021). The heterogeneous climatic conditions in the Gaoligong Mountains have produced many plant species with different ecological adaptation strategies (Chen et al., 2018), and thus the region is well suited for studying variation in SDS along elevational gradients.

### 2.2 Seed collection

**FIGURE 1:**
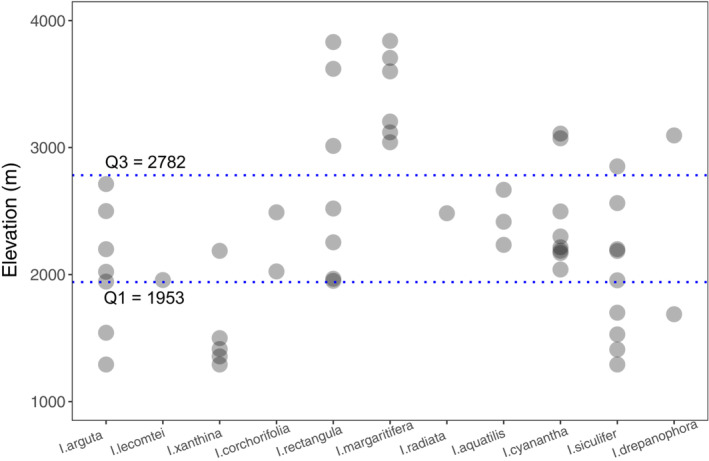
Elevational distribution of seed provenance localities. Each point represents a population of *Impatiens* species. Two dashed lines representing 25^th^ and 75^th^ quantiles of elevational distribution serve as low‐ and high‐elevation boundaries, respectively.

From August 2021 to October 2021, mature seeds from 51 populations of 11 *Impatiens* species were collected at different elevations in the Gaoligong Mountains. Global positioning system (GPS) coordinates of population provenance were also recorded using a handheld GPS device. The provenance localities were at elevations ranging from 1292 to 3840 m a.s.l., covering much of the elevational range of the genus (Figure 1). For each population, seeds were collected from up to 10 individuals at the beginning of the dispersal period when all seeds were mature. Seeds were packed in paper envelopes and transported to the laboratory, where they were spread on tables (16–22°C and 45%–50% relative humidity) for 2 days, followed by manual processing to remove all impurities and visibly damaged seeds.

### 2.3 Determination of SDS

Seed dormancy is generally defined as the failure of a seed to germinate (or a low percentage of germination) despite suitable environmental conditions. Most fresh seeds are in a state of dormancy at maturity (Baskin & Baskin, 2014). Therefore, dormancy percentage of fresh seeds can be used to measure SDS, calculated as SDS = dormant seeds/(dormant seeds + germinated seeds).

Five days after harvest, the collected *Impatiens* seeds were sown on five layers of damp filter paper in 90‐mm diameter Petri dishes. The dishes were placed in light‐controlled incubators (12‐h daily photoperiod) at three constant temperatures (15, 20 and 25°C) and regularly watered with distilled water (Veselá et al., 2020). As seeds collected at different elevations may germinate at different optimum temperatures, we selected the three above temperatures to approximate optimum germination temperatures of seeds from high to low elevations, respectively (Veselá et al., 2020). Each population consisted of two replicates of 30 seeds at each temperature treatment. Although there were insufficient mature seeds for three replicates, each population contained 180 seeds. Germination was scored daily and seeds with visible radicles (about 2 mm) were considered germinated. All trials were conducted for 4 weeks, until no further germination was observed. Nongerminated seeds were cut with a scalpel, and those showing complete and hard embryos were considered viable (Aragón‐Gastélum et al., 2018). The percentage of dormant seeds during each trial was defined as the nongerminated percentage obtained after discarding nonviable and moldy seeds. For comprehensive quantification of seed dormancy, average dormancy percentage at the three temperatures was used to measure the SDS of each population. However, to assess consistency of results at different temperatures, dormancy data obtained at the three temperatures were analyzed separately (Table S1 and Figure S1). We did not measure the pure effects of genetic differences in species on SDS as we did not obtain sufficient seeds from common garden experiments due to the extremely low survival and seed‐setting rates of *Impatiens* plants.

### 2.4 Climatic data

Nineteen global bioclimatic layers (Bio1–19, Table 1) were downloaded from WorldClim v.2.1 with a spatial resolution of 1 km^2^ (average for the years 1970–2000) (Fick & Hijmans, 2017). For each *Impatiens* population, bioclimatic variables were obtained from the bioclimatic layers according to the geographic coordinates of the population using the “extract values to points” function in ArcGIS v.10.2 (ESRI, Redlands, CA, USA).

**TABLE 1:** Correlations between SDS and 19 bioclimatic variables estimated for seed collection sites of *Impatiens* species.

Bioclimatic variable	Spearman *r*	*p*‐value
**Annual mean temperature (Bio1)**	**−.312**	**.026**
**Mean diurnal range (Bio2)**	**−.343**	**.014**
**Isothermality (Bio3)**	**−.362**	**.009**
**Temperature seasonality (Bio4)**	**.372**	**.007**
Max temperature of warmest month (Bio5)	−.206	.146
**Min temperature of coldest month (Bio6)**	**−.338**	**.015**
**Temperature annual range (Bio7)**	**.364**	**.009**
Mean temperature of wettest quarter (Bio8)	−.247	.081
**Mean temperature of driest quarter (Bio9)**	**−.286**	**.041**
Mean temperature of warmest quarter (Bio10)	−.247	.081
**Mean temperature of coldest quarter (Bio11)**	**−.374**	**.007**
Annual precipitation (Bio12)	.065	.65
Precipitation of wettest month (Bio13)	.065	.649
Precipitation of driest month (Bio14)	−.029	.843
Precipitation seasonality (Bio15)	−.068	.633
Precipitation of wettest quarter (Bio16)	.041	.774
Precipitation of driest quarter (Bio17)	−.206	.146
Precipitation of warmest quarter (Bio18)	.041	.774
Precipitation of coldest quarter (Bio19)	.202	.154

*Note*: Statistically significant variables (*p* < .05) are in bold.

### 2.5 Phylogenetic tree construction

Nuclear ribosomal internal transcribed spacer (ITS) and chloroplast DNA (*atpB*‐*rbcL* and *trnL‐F*) have been shown to be valuable in phylogenetic studies within Balsaminaceae (Yu et al., 2016). Therefore, all available ITS, *atpB*‐*rbcL*, and *trnL‐F* sequences of the 11 *Impatiens* species were downloaded from the GenBank nucleotide database (Table S2). All sequences were aligned separately by class using default parameters in MEGA v.10.2 to obtain three sequence alignments (i.e., ITS, *atpB*‐*rbcL*, and *trnL‐F*). The three sequence alignments were subsequently concatenated into a single alignment using Geneious v.9.1. Based on the concatenated alignment, a phylogenetic tree was constructed using the maximum‐likelihood approach in RAxML‐NG with default parameters and a constrained topology in Newick format (Yu et al., 2016) in the CIPRES Science Gateway (https://www.phylo.org/).

### 2.6 Statistical analyses

The dormancy data were not normalized. Therefore, nonparametric analysis of variance (nonparametric ANOVA) with the Kruskal–Wallis test was used to assess dormancy percentage differences among species using the “kruskal.test” function in the *stats* package in R. As closely related species tend to have similar SDS (Felsenstein, 1985), Blomberg's *K* was calculated as an index of phylogenetic conservatism. Any *K* value significantly higher than zero can be regarded as trait evolution approaching Brownian motion to varying degrees (Chen et al., 2022). This metric was calculated using the “phylosig” function in the R package *phytools* with arcsine‐transformed mean dormancy percentages of all populations for each species (Baskin & Baskin, 2014). To study the effects of phylogeny and elevation on SDS, we applied a logistic regression model (LRM) assuming a quasi‐binomial distribution and a logit link function using the “gls” function in the R package *nlme*. For the LRM, the phylogenetic tree was transformed into phylogenetic distance (using *I. siculifer* as a baseline) as an independent variable using the “cophenetic” function in the R package *stats*, and all independent variables were standardized by subtracting the mean and dividing by the standard deviation.

Considering the correlations among the 19 bioclimatic variables, hierarchical partitioning analysis, which is a robust regression for a set of collinear predictors, was performed using the “hier.part” function in the *hier.part* package to assess the relative importance of each climatic variable (Huang et al., 2016). Before analysis, bioclimatic variables not significantly correlated with SDS were removed to reduce dataset complexity, with eight variables finally retained (i.e., Bio1–4, Bio6–7, Bio9, and Bio11) (Table 1). To identify climatic factors driving seed dormancy variation with elevation, three steps were performed: (1) We built a LRM with dormancy percentage as a dependent variable and Bio1–4, Bio6–7, Bio9, and Bio11 as independent variables, (2) we selected the best model (i.e., SDS = *β*_0_ + *β*_1_Bio1 + *β*_2_Bio9) from step 1 using the “glmulti” function in the *glmulti* package, and (3) we calculated Spearman rank correlations of the residuals of the best model with phylogenetic distance and elevation using the “cor.test” function in the *stats* package. All statistical analyses were conducted using R v.4.0.2 (R Core Team, 2020).

## 3 RESULTS

### 3.1 Phylogenetic signals of SDS in *Impatiens* species

**FIGURE 2:**
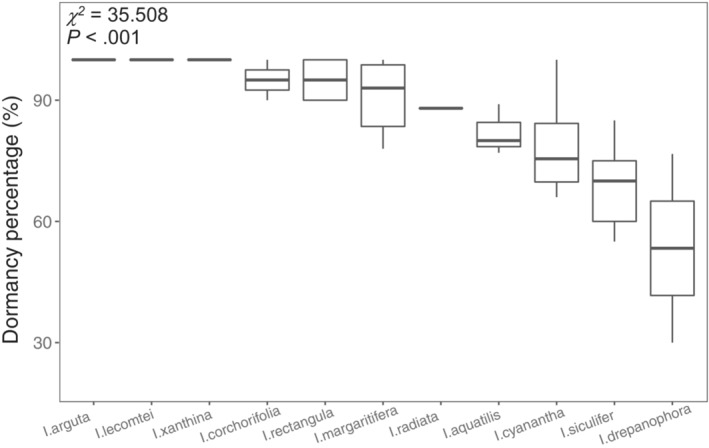
Distribution of seed dormancy percentages in *Impatiens* species. Boxes span from 25^th^ to 75^th^ quantiles, with bold line representing median, and whiskers span from 5^th^ to 95^th^ quantiles. Chi‐square value shows significant differences in seed dormancy percentages among *Impatiens* species based on Kruskal‐Wallis test (non‐parametric ANOVA).

**FIGURE 3:**
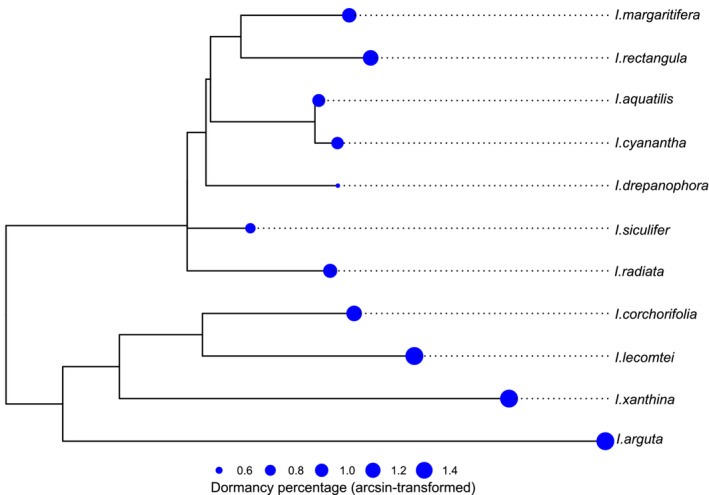
Phylogenetic tree of 11 *Impatiens* species with arcsine‐transformed mean dormancy percentages. Size of dots is proportional to SDS. Phylogenetic tree was constructed based on three DNA sequences (ITS, *atpB‐rbcL*, and *trnL‐F*).

Significant differences were detected in the proportion of dormant seeds among *Impatiens* species (*χ* ^*2*^ = 35.508, *df* = 10, *p* < .001; Figure 2), with the lowest proportion found in *I. drepanophora* (mean = 0.534, SD = 0.33), and no germinants found in *I. arguta*, *I. lecomtei*, or *I. xanthina* (Figure 2). We also detected a significant phylogenetic signal in the SDS of *Impatiens* species (*K* = 1.068, *p* = .002; Figure 3), and the proportions of dormant seeds clustered together across the phylogeny tree (Figure 3).

### 3.2 Effects of phylogeny and elevation on SDS of *Impatiens* species

**FIGURE 4:**
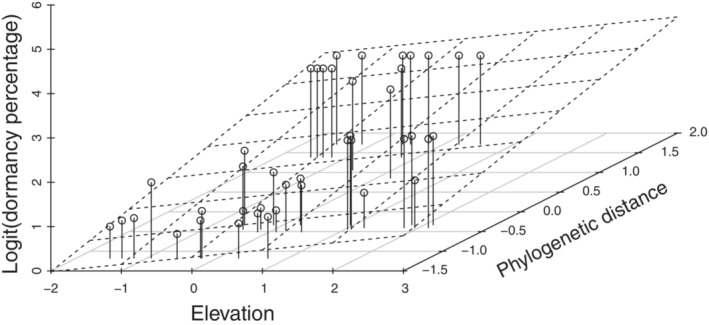
Effects of phylogenetic distance and elevation on SDS. Dotted line represents regression plane. Both phylogenetic distance and elevation were standardized by subtracting the mean and dividing by the standard deviation.

The LRM results showed that phylogenetic distance and elevation were positively associated with *Impatiens* seed dormancy (effect size = 1.337, *p* < .001; effect size = 0.518, *p* = .001, respectively; Figure 4). Furthermore, phylogenetic distance and elevation accounted for 63.629% of the total variation in the SDS of *Impatiens* populations (Figure 4).

### 3.3 Effects of climate on SDS variation

Based on the best LRM of seed dormancy proportion (*χ* ^2^ = 0.531, *df* = 8, *p* > .5), Bio1 (annual mean temperature) was negatively associated with SDS (effect size = −7.059, *p* = .053; Table 2), while Bio9 (mean temperature of driest quarter) was positively associated with SDS (effect size = 6.544, *p* = .076; Table 2). Hierarchical partitioning analysis revealed that Bio1 independently explained 20.768% of the variance in SDS, while Bio9 explained 21.187% (Table 2). Conversely, the relationships between SDS and precipitation variables were extremely weak (*p* > .1; Table 1). Therefore, seed dormancy proportion in *Impatiens* species was negatively correlated with heat, but not affected by moisture in the mountain regions of southwest China (Table 1 and Table 2).

**TABLE 2:** Effects of climatic variables on SDS.

Independent variable	Estimate ± SE	*t*‐value	*p*‐value	Independent percentage
Intercept	1.968 ± 0.191	10.311	<.001	
Bio1	−7.059 ± 3.563	−1.981	.053	20.768%
Bio9	6.544 ± 3.604	1.816	.076	21.187%

*Note*: Bio1 = annual mean temperature, Bio9 = mean temperature of driest quarter. Columns two to four showing estimates, *t*‐values and *p*‐values are the results from the best logistic regression model with quasi‐binomial distribution used to determine effects of climatic variables on SDS. This model well fit the *Impatiens* seed dormancy data (*χ* ^2^ = 0.531, *df* = 8, *p* > .5). The last column showing independent percentage of variance explained by each variable is the results of hierarchical partitioning analysis.

**FIGURE 5:**
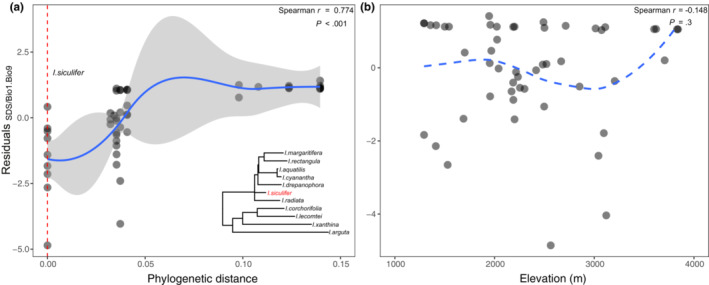
Spearman correlations of Bio1 and Bio9 residuals on SDS (R_SDS/Bio1.Bio9_) with phylogeny (a) and elevation (b). Red dashed line in (a) represents *I. siculifer* as the baseline for phylogenetic distances and blue trend line shows significant correlation predictions and associated 95% confidence bands. Bottom right of (a) is phylogenetic tree of 11 *Impatiens* species. Blue dashed line in (b) indicates correlation between R_SDS/Bio1.Bio9_ and elevation is not significant.

The residuals of Bio1 and Bio9 on SDS (i.e., R_SDS/Bio1.Bio9_) were significantly correlated with phylogeny (Spearman *r* = .774, *S* = 4993.2, *p* < .001; Figure 5a), whereas R_SDS/Bio1.Bio9_ showed a nonsignificant relationship with elevation (Spearman *r* = −.148, *S* = 25,370, *p* = .3; Figure 5b).

## 4 DISCUSSION

### 4.1 Phylogenetic conservatism of SDS in *Impatiens* species

We detected significant variation in seed dormancy proportion and strong phylogenetic signals in *Impatiens* species, providing evidence that closely related species exhibit more similar SDS than distantly related species. Phylogenetic constraints on seed dormancy may be explained by related species experiencing similar evolutionary histories and natural selection pressures, resulting in similar SDS and inherited seed dormancy. Our results are consistent with previous studies showing that phylogeny is a limiting factor of dormancy‐related traits. For example, Seglias et al. (2018) reported that SDS is significantly correlated with phylogenetic relatedness in eight forb species in southwest USA, and Carta et al. (2016) reported that phylogenetically related *Romulea* species (Iridaceae) in the Mediterranean show similar requirements for seed dormancy release. Here, seed dormancy of closely related *Impatiens* species showed a similar response to changing environments due to the phylogenetic constraints on plant seed dormancy.

### 4.2 Effects of phylogeny and elevation on SDS variation in *Impatiens* species

Our results indicated that phylogeny is a more important factor than elevation in shaping seed dormancy variation in *Impatiens* species. This suggests that genetics may be the most important factor influencing SDS variation in *Impatiens* species in the mountains of southwest China, corresponding to the significant phylogenetic signal of SDS. Evidence suggests that seed dormancy is an adaptive trait that depends on the cumulative action of many genes (Batlla & Benech‐Arnold, 2007; Bentsink et al., 2006), which may result in continuous variance in seed dormancy. However, it is not clear whether genetically restricted seed dormancy can be generalized to other plant taxa and geographic regions, and thus further studies are required.

After controlling for phylogeny, a strong positive relationship was found between elevation and fresh seed dormancy percentage in *Impatiens* species. These results suggest the existence of an elevational pattern in SDS variation, which may be explained by transmission of elevational cues to seeds by mother plants distributed across an elevational gradient, resulting in variations in SDS with elevation (Penfield, 2017). The impact of parental elevational cues on seed dormancy also varies with elevation due to the increase in environmental fluctuations (Lampei et al., 2017). This variation in parental effects with elevation assists seedlings in adapting to different habitats by selecting suitable germination time regulated by SDS (Lampei et al., 2017). As an adaptive trait, deep seed dormancy appears to promote plant survival in high‐elevation habitats (Baskin & Baskin, 2014). Consistently, *S. longifolia* populations contain higher proportions of dormant seeds at high elevations (Cotado et al., 2020), *Physalis* species show an increase in dormant seeds with increasing elevation (Farooq et al., 2021), and *C. halicacabum* seeds show more complex seed dormancy at high elevations than at low elevations (Thusithana et al., 2021). These results suggest that natural selection pressure should favor plants that produce more dormant mature seeds at higher elevations. However, it should be noted that due to the lack of successful common garden experiments, we were unable to measure the pure effects of genetic differences in species on SDS. As a result, the variance of SDS explained by elevation included both a pure fraction and a common fraction with intraspecific genetic differences. Nevertheless, our results contribute to the limited research on the combined effects of phylogeny and elevation on SDS variation (Seglias et al., 2018) and provide new insights into the effects of phylogeny and elevation on seed dormancy in mountain ecosystems.

### 4.3 Effects of climate on SDS variation along elevation

We found a significant relationship between SDS and temperature, indicating that heat is a dominant climatic factor influencing seed dormancy. This result is consistent with previous research showing that small differences in temperature can greatly impact seed dormancy variation (Penfield, 2017). Frost‐tolerant species tend to produce small seeds with a high degree of dormancy under low‐temperature stress, thus facilitating plant adaptation to the extended cold periods typical of high elevation habitats (Rosbakh et al., 2022). While other species (e.g., *Cistus*) grown in high‐temperature habitats require higher heat to release seed dormancy (Zomer et al., 2022), this requirement may prevent seeds in low‐land regions from germinating during occasional warm winter days. The effect of parental temperature‐imposed dormancy on seeds can vary across species. For example, *Pennisetum typhoides* and *Arabidopsis thaliana* exhibit limited responses to temperatures exceeding 22°C but show significant increases in seed dormancy at temperatures of 19°C and 15°C, respectively (Fenner, 1991; He et al., 2016). Unlike temperature, our results showed that precipitation was not significantly associated with seed dormancy in *Impatiens*, inconsistent with previous research showing an increase in dormant seeds (e.g., *C. halicacabum*) with decreasing precipitation (Thusithana et al., 2021). This may be because *Impatiens* germination coincides with the rainy season in southwest China, and moisture is therefore not a limiting factor for seed development. Heat and moisture properties during seed maturation are “remembered” by seeds through the mother plant, known as maternal effects, and can shape SDS.

Our results showed a nonsignificant relationship between R_SDS/Bio1.Bio9_ (i.e., residuals of SDS on Bio1 and Bio9) and elevation, suggesting that climatic factors drive the elevational pattern of SDS variation in *Impatiens* species in the mountains of southwest China. This finding is consistent with previous research showing that soil temperature drives the allocation of reproductive biomass along elevation (Chen et al., 2020). Seed dormancy is thought to be closely related to environmental heterogeneity and changes in climatic factors with increasing elevation (Cotado et al., 2020; Jurado & Flores, 2005). The harsh climatic changes imposed by higher elevation (e.g., short growing season, low temperature, and fluctuating precipitation) provide considerable natural selection pressure on seed plants in mountain ecosystems, leading to increased seed dormancy as an adaptive trait with increasing elevation. According to our results, seeds in the high‐elevation mountainous regions of southwest China must remain dormant for longer periods to avoid germination during adverse conditions that are not conducive for seedling growth. Climate change is expected to negatively impact biodiversity at high elevations in the mountains of southwest China (Xu et al., 2009). However, seed dormancy may contribute to the ability of plants to persist under changing environments. As such, high‐elevation plant diversity may not be as negatively impacted as predicted.

We also observed a significant relationship between R_SDS/Bio1.Bio9_ and phylogenetic distance, indicating that the effect of phylogeny on SDS is independent of current climate. As speciation and extinction occur over evolutionary timescales of hundreds of millions of years (Svenning et al., 2015), phylogeny is shaped by paleoclimate rather than present‐day climate. Empirical evidence has also shown that evolution of seed dormancy is significantly correlated with paleotemperature (Zhang et al., 2022).

## 5 CONCLUSIONS

This study provides evidence that phylogeny and elevation are important factors shaping seed dormancy in *Impatiens* species in the mountains of southwest China. Results showed that closely related species exhibited more similar SDS than distantly related species. Furthermore, high‐elevation species produced more dormant seeds than low‐elevation species as an adaptation to harsher habitats. Heat was the main driver of SDS variation with elevation, while the effect of phylogeny on SDS was independent of climate. In this study, we not only quantified the effects of phylogeny on SDS but also disentangled the phylogenetic and ecological determinants of seed dormancy in mountainous ecosystems. Seed dormancy is an ecological adaptation strategy and is phylogenetically conserved, thus our findings provide new insights into the response of seed plant diversity to climate change.

## AUTHOR CONTRIBUTIONS

**Kai Chen:** Conceptualization (lead); data curation (equal); investigation (equal); writing – original draft (lead). **Zi‐Hong Chen:** Data curation (equal); writing – review and editing (equal). **Yuan‐Yuan Huang:** Data curation (equal); investigation (equal). **Zhong‐Hua Jiang:** Data curation (equal); investigation (equal).

## CONFLICT OF INTEREST STATEMENT

The authors declare no conflicts of interest.

## Supporting information


Appendix S1.

## Data Availability

The data that support the findings of this study is accessible at the Dryad Digital Repository (DOI): 10.5061/dryad.gqnk98st8.

## References

[ece39986-bib-0001] Allen, P. S. , & Meyer, S. E. (1998). Ecological aspects of seed dormancy loss. Seed Science Research, 8(2), 183–191. 10.1017/S0960258500004098

[ece39986-bib-0002] Andersson, L. , & Milberg, P. (1998). Variation in seed dormancy among mother plants, populations and years of seed collection. Seed Science Research, 8(1), 29–38. 10.1017/S0960258500003883

[ece39986-bib-0003] Aragón‐Gastélum, J. L. , Flores, J. , Jurado, E. , Ramírez‐Tobías, H. M. , Robles‐Díaz, E. , Rodas‐Ortiz, J. P. , & Yáñez‐Espinosa, L. (2018). Potential impact of global warming on seed bank, dormancy and germination of three succulent species from the Chihuahuan Desert. Seed Science Research, 28(4), 312–318. 10.1017/S0960258518000302

[ece39986-bib-0004] Baskin, C. C. , & Baskin, J. M. (2014). Seed ecology, biogeography, and evolution of dormancy and germination. New York Press.

[ece39986-bib-0005] Baskin, J. M. , & Baskin, C. C. (2004). A classification system for seed dormancy. Seed Science Research, 14(1), 1–16. 10.1079/SSR2003150

[ece39986-bib-0006] Batlla, D. , & Benech‐Arnold, R. L. (2007). Predicting changes in dormancy level in weed seed soil banks: Implications for weed management. Crop Protection, 26(3), 189–197. 10.1016/j.cropro.2005.07.014

[ece39986-bib-0007] Bentsink, L. , Jowett, J. , Hanhart, C. J. , & Koornneef, M. (2006). Cloning of *DOG1*, a quantitative trait locus controlling seed dormancy in *Arabidopsis* . Proceedings of the National Academy of Sciences of the United States of America, 103(45), 17042–17047. 10.1073/pnas.0607877103 17065317 PMC1636575

[ece39986-bib-0008] Carta, A. , Hanson, S. , & Müller, J. V. (2016). Plant regeneration from seeds responds to phylogenetic relatedness and local adaptation in Mediterranean *Romulea* (Iridaceae) species. Ecology and Evolution, 6(12), 4166–4178. 10.1002/ece3.2150 27516872 PMC4884198

[ece39986-bib-0009] Chen, K. , Burgess, K. S. , He, F. , Yang, X. Y. , Gao, L. M. , & Li, D. Z. (2022). Seed traits and phylogeny explain plants' geographic distribution. Biogeosciences, 19(19), 4801–4810. 10.5194/bg-19-4801-2022

[ece39986-bib-0010] Chen, K. , Burgess, K. S. , Yang, X. Y. , Luo, Y. H. , Gao, L. M. , & Li, D. Z. (2018). Functional trade‐offs and the phylogenetic dispersion of seed traits in a biodiversity hotspot of the mountains of Southwest China. Ecology and Evolution, 8(4), 2218–2230. 10.1002/ece3.3805 29468038 PMC5817125

[ece39986-bib-0011] Chen, K. , Liu, Q. , Chen, Z. H. , & Li, Z. L. (2020). Soil temperature drives elevational patterns of reproductive allometry in a biodiversity hotspot. Plant Ecology, 221, 979–988. 10.1007/s11258-020-01055-8

[ece39986-bib-0012] Cotado, A. , Garcia, M. B. , & Munné‐Bosch, S. (2020). Physiological seed dormancy increases at high altitude in Pyrenean saxifrage (*Saxifraga longifolia* Lapeyr.). Environmental and Experimental Botany, 171, 103929. 10.1016/j.envexpbot.2019.103929

[ece39986-bib-0013] Donohue, K. , Dorn, L. , Griffith, C. , Kim, E. , Aguilera, A. , Chandra, R. , & Schmitt, J. (2005). Niche construction through germination cueing: Life‐history responses to timing of germination in *Arabidopsis thaliana* . Evolution, 59(4), 771–785. 10.1554/04-655 15926688

[ece39986-bib-0014] Farooq, S. , Onen, H. , Ozaslan, C. , El‐Shehawi, A. M. , & Elseehy, M. M. (2021). Characteristics and methods to release seed dormancy of two ground cherry (*physalis*) species. Journal of Applied Research on Medicinal and Aromatic Plants, 25, 100337. 10.1016/j.jarmap.2021.100337

[ece39986-bib-0015] Felsenstein, J. (1985). Phylogenies and the comparative method. The American Naturalist, 125(1), 1–15. 10.1086/284325

[ece39986-bib-0016] Fenner, M. (1991). The effects of the parent environment on seed germinability. Seed Science Research, 1(2), 75–84. 10.1017/S0960258500000696

[ece39986-bib-0017] Fenner, M. (Ed.). (2002). Seeds: The ecology of regeneration in plant communities (2nd ed.). Oxford University Press.

[ece39986-bib-0018] Fick, S. E. , & Hijmans, R. J. (2017). WorldClim 2: New 1‐km spatial resolution climate surfaces for global land areas. International Journal of Climatology, 37(12), 4302–4315. 10.1002/joc.5086

[ece39986-bib-0019] Finch‐Savage, W. E. , & Leubner‐Metzger, G. (2006). Seed dormancy and the control of germination. New Phytologist, 171(3), 501–523. 10.1111/j.1469-8137.2006.01787.x 16866955

[ece39986-bib-0020] Ge, W. , Bu, H. , Wang, X. , Martinez, S. A. , & Du, G. (2020). Inter‐ and intra‐specific difference in the effect of elevation and seed mass on germinability of eight *allium* species. Global Ecology and Conservation, 22, e01016. 10.1016/j.gecco.2020.e01016

[ece39986-bib-0021] He, H. , Willems, L. A. J. , Batushansky, A. , Fait, A. , Hanson, J. , Nijveen, H. , Hilhorst, H. W. M. , & Bentsink, L. (2016). Effects of parental temperature and nitrate on seed performance are reflected by partly overlapping genetic and metabolic pathways. Plant and Cell Physiology, 57(3), 473–487. 10.1093/pcp/pcv207 26738545

[ece39986-bib-0022] Huang, Z. , Liu, S. , Bradford, K. J. , Huxman, T. E. , & Venable, D. L. (2016). The contribution of germination functional traits to population dynamics of a desert plant community. Ecology, 97(1), 250–261. 10.1890/15-0744.1 27008793

[ece39986-bib-0023] Jurado, E. , & Flores, J. (2005). Is seed dormancy under environmental control or bound to plant traits? Journal of Vegetation Science, 16(5), 559–564. 10.1111/j.1654-1103.2005.tb02396.x

[ece39986-bib-0024] Lampei, C. , Metz, J. , & Tielbörger, K. (2017). Clinal population divergence in an adaptive parental environmental effect that adjusts seed banking. New Phytologist, 214(3), 1230–1244. 10.1111/nph.14436 28152187

[ece39986-bib-0025] Liang, D. , Pan, X. , Luo, X. , Wenda, C. , Zhao, Y. , Hu, Y. , Robinson, S. K. , & Liu, Y. (2021). Seasonal variation in community composition and distributional ranges of birds along a subtropical elevation gradient in China. Diversity and Distribution, 27(12), 2527–2541. 10.1111/ddi.13420

[ece39986-bib-0026] Moles, A. T. , Ackerly, D. D. , Webb, C. O. , Tweddle, J. C. , Dickie, J. B. , Pitman, A. J. , & Westoby, M. (2005). Factors that shape seed mass evolution. Proceedings of the National Academy of Sciences of the United States of America, 102(30), 10540–10544. 10.1073/pnas.0501473102 16030149 PMC1180762

[ece39986-bib-0027] Orrù, M. , Mattana, E. , Pritchard, H. W. , & Bacchetta, G. (2012). Thermal thresholds as predictors of seed dormancy release and germination timing: Altitude‐related risks from climate warming for the wild grapevine *Vitis vinifera* subsp. *sylvestris* . Annals of Botany, 110(8), 1651–1660. 10.1093/aob/mcs218 23071219 PMC3503498

[ece39986-bib-0028] Penfield, S. (2017). Seed dormancy and germination. Current Biology, 27(17), R874–R878. 10.1016/j.cub.2017.05.050 28898656

[ece39986-bib-0029] R Core Team . (2020). R: A language and environment for statistical computing. R Foundation for Statistical Computing. https://www.R‐project.org/

[ece39986-bib-0030] Rosbakh, S. , Chalmandrier, L. , Phartyal, S. , & Poschlod, P. (2022). Inferring community assembly processes from functional seed trait variation along elevation gradient. Journal of Ecology, 110(10), 2374–2387. 10.1111/1365-2745.13955

[ece39986-bib-0031] Seglias, A. E. , Williams, E. , Bilge, A. , & Kramer, A. T. (2018). Phylogeny and source climate impact seed dormancy and germination of restoration‐relevant forb species. PLoS One, 13(2), e0191931. 10.1371/journal.pone.0191931 29401470 PMC5798788

[ece39986-bib-0032] Sundqvist, M. K. , Sanders, N. J. , & Wardle, D. A. (2013). Community and ecosystem responses to elevational gradients: Processes, mechanisms, and insights for global change. Annual Review of Ecology, Evolution, and Systematics, 44, 261–280. 10.1146/annurev-ecolsys-110512-135750

[ece39986-bib-0033] Svenning, J. C. , Eiserhardt, W. L. , Normand, S. , Ordonez, A. , & Sandel, B. (2015). The influence of paleoclimate on present‐day patterns in biodiversity and ecosystems. Annual Review of Ecology, Evolution, and Systematics, 46, 551–572. 10.1146/annurev-ecolsys-112414-054314

[ece39986-bib-0034] Thusithana, V. , Amarasekara, R. W. K. , Gehan Jayasuriya, K. M. G. , Gama‐Arachchige, N. S. , Baskin, C. C. , & Baskin, J. M. (2021). Seed dormancy of *Cardiospermum halicacabum* (Sapindaceae) from three precipitation zones in Sri Lanka. Plant Biology, 23(1), 148–155. 10.1111/plb.13189 32989855

[ece39986-bib-0035] Veselá, A. , Dostálek, T. , Rokaya, M. B. , & Münzbergová, Z. (2020). Seed mass and plant home site environment interact to determine alpine species germination patterns along an elevation gradient. Alpine Botany, 130, 101–113. 10.1007/s00035-020-00242-7

[ece39986-bib-0036] Wagmann, K. , Hautekèete, N. C. , Piquot, Y. , Meunier, C. , Schmitt, S. E. , & Van Dijk, H. (2012). Seed dormancy distribution: Explanatory ecological factors. Annals of Botany, 110(6), 1205–1219. 10.1093/aob/mcs194 22952378 PMC3478053

[ece39986-bib-0037] Willis, C. G. , Baskin, C. C. , Baskin, J. M. , Auld, J. R. , Venable, D. L. , Cavender‐Bares, J. , Donohue, K. , & Rubio de Casas, R. (2014). The evolution of seed dormancy: Environmental cues, evolutionary hubs, and diversification of the seed plants. New Phytologist, 203(1), 300–309. 10.1111/nph.12782 24684268

[ece39986-bib-0038] Xu, J. C. , Grumbine, R. E. , Shrestha, A. , Eriksson, M. , Yang, X. , Wang, Y. , & Wilkes, A. (2009). The melting Himalayas: Cascading effects of climate change on water, biodiversity, and livelihoods. Conservation Biology, 23(3), 520–530. 10.1111/j.1523-1739.2009.01237.x 22748090

[ece39986-bib-0039] Yu, S. X. , Janssens, S. B. , Zhu, X. Y. , Lidén, M. , Gao, T. G. , & Wang, W. (2016). Phylogeny of *impatiens* (Balsaminaceae): Integrating molecular and morphological evidence into a new classification. Cladistics, 32(2), 179–197. 10.1111/cla.12119 34732016

[ece39986-bib-0040] Zhang, Y. , Liu, Y. , Sun, L. , Baskin, C. C. , Baskin, J. M. , Cao, M. , & Yang, J. (2022). Seed dormancy in space and time: Global distribution, paleoclimatic and present climatic drivers, and evolutionary adaptations. New Phytologist, 234(5), 1770–1781. 10.1111/nph.18099 35292965

[ece39986-bib-0041] Zomer, M. , Moreira, B. , & Pausas, J. G. (2022). Fire and summer temperatures interact to shape seed dormancy thresholds. Annals of Botany, 129(7), 809–816. 10.1093/aob/mcac047 35390121 PMC9292603

