# Supplementary material for: Elevation and phylogeny shape herbaceous seed dormancy in a biodiversity hotspot of southwest China
Source: Ecol Evol. 2023 Apr 18;13(4):e9986. doi: 10.1002/ece3.9986 (PMC10111168; doi:10.1002/ece3.9986)
Supplement: Supplementary file 1 — Appendix S1. [file ECE3-13-e9986-s001.doc]

**Appendix**

Table S1 Phylogenetic signals of SDS at three different germination temperatures.

| Germination temperature | Blomberg’s *K* | *P*-value |
| --- | --- | --- |
| 15 ℃ | 0.580 | > 0.05 |
| 20 ℃ | 1.144 | < 0.01 |
| 25 ℃ | 1.212 | < 0.01 |

Table S2 GenBank accession numbers for sequences used to construct phylogenetic tree in this study.

| Species | GenBank accession number | | |
| --- | --- | --- | --- |
|  | ITS | *atpB-rbcL* | *trnL-F* |
| *I. aquatilis* | AY348745 | DQ147811 | KP776115 |
| *I. arguta* | AY348746 | DQ147812 | KP776116 |
| *I. corchorifolia* | AY348767 | DQ147831 | KP776127 |
| *I. cyanantha* | AY348770 | DQ147833 |  |
| *I. drepanophora* | AY348776 | DQ147838 |  |
| *I. lecomtei* | AY348802 | DQ147855 |  |
| *I. margaritifera* | KP776084 | KP776036 |  |
| *I. radiata* | AY348824 | KP776047 | KP776160 |
| *I. rectangula* | AY348825 | DQ147874 |  |
| *I. siculifer* | KP776101 | KP776049 |  |
| *I. xanthina* | AY348850 | DQ147893 |  |
| *Hydrocera triflora* (outgroup) | AY348853 | DQ147895 |  |





Figure S1 Relative importance of climatic variables to SDS at three germination temperatures (15 ℃, 20 ℃, and 25 ℃). Column represents independent percentage of variance explained by each variable relative to the others. Full names of the eight variables are shown in Table 1.
